# THETA-Rhythm Makes the World Go Round: Dissociative Effects of TMS Theta Versus Alpha Entrainment of Right pTPJ on Embodied Perspective Transformations

**DOI:** 10.1007/s10548-017-0557-z

**Published:** 2017-03-03

**Authors:** Gerard Gooding-Williams, Hongfang Wang, Klaus Kessler

**Affiliations:** Aston Brain Centre, Aston University, Birmingham, B4 7ET UK

**Keywords:** Transcranial magnetic stimulation, Rhythmic entrainment, Tehta oscillations, Temporoparietal junction, Perspective takin, Embodiment

## Abstract

Being able to imagine another person’s experience and perspective of the world is a crucial human ability and recent reports suggest that humans “embody” another’s viewpoint by mentally rotating their own body representation into the other’s orientation. Our recent Magnetoencephalography (MEG) data further confirmed this notion of embodied perspective transformations and pinpointed the right posterior temporo-parietal junction (pTPJ) as the crucial hub in a distributed network oscillating at theta frequency (3–7 Hz). In a subsequent transcranial magnetic stimulation (TMS) experiment we interfered with right pTPJ processing and observed a modulation of the embodied aspects of perspective transformations. While these results corroborated the role of right pTPJ, the notion of theta oscillations being the crucial neural code remained a correlational observation based on our MEG data. In the current study we therefore set out to confirm the importance of theta oscillations directly by means of TMS entrainment. We compared entrainment of right pTPJ at 6 Hz vs. 10 Hz and confirmed that only 6 Hz entrainment facilitated embodied perspective transformations (at 160° angular disparity) while 10 Hz slowed it down. The reverse was true at low angular disparity (60° between egocentric and target perspective) where a perspective transformation was not strictly necessary. Our results further corroborate right pTPJ involvement in embodied perspective transformations and highlight theta oscillations as a crucial neural code.

**Electronic supplementary material:**

The online version of this article (doi:10.1007/s10548-017-0557-z) contains supplementary material, which is available to authorized users.

## Introduction

Being able to imagine another person’s experience and perspective of the world is a crucial human social ability (Call and Tomasello 1999) and recent reports suggest that humans “embody” another’s viewpoint by mentally rotating their own body representation into the other’s orientation (Kessler et al. 2014; Kessler and Rutherford 2010; Kessler and Thomson 2010; Surtees et al. 2013). Using Magnetoencephalography (MEG) our group recently reported that brain oscillations at theta frequency (3–7 Hz), originating from the right posterior temporo-parietal-junction (pTPJ) reflected cognitive as well as embodied processing elements of perspective transformations (Wang et al. 2016, Expt 1). This was subsequently confirmed using transcranial magnetic stimulation (TMS; Wang et al. 2016, Expt 2) of right pTPJ with a dual pulse protocol (dpTMS), which affected embodied aspects of perspective taking (conforming to other stimulation reports, e.g., Blanke et al. 2005; Santiesteban et al. 2012; van Elk et al. 2016). Note that especially the posterior part of the right TPJ (pTPJ) has recently been proposed as a subsection associated with social processing, whereas more anterior parts may be more involved in attention and stimulus-related processing (e.g. Carter and Huettel 2013; Igelström et al. 2015).

Overall these findings emphasised theta oscillations originating from right pTPJ as the core of a wide-spread cortical network involved in embodying another’s viewpoint. However, while dpTMS was suitable for revealing the involvement of right pTPJ, it was insufficient for corroborating the importance of theta oscillations. We therefore employed frequency-specific TMS entrainment (Romei et al. 2016; Ruzzoli and Soto-Faraco 2014; Thut and Miniussi 2009) in the current study to specifically test the hypothesis that theta entrainment of right pTPJ supports visuospatial perspective taking, resulting in faster response times (RTs), while alpha entrainment might inhibit and slow down visuospatial perspective taking. The latter was based on the general notion that increased alpha amplitudes reflect inhibition (Jensen and Mazaheri 2010; Klimesch et al. 2007) as well as on our observation in Wang et al. (2016) that theta and alpha oscillations fulfil complementary roles, with alpha supporting visual processing at 60°, while theta supporting full-blown visuospatial perspective taking at 160°.

## Materials and Methods

### Participants

Fourteen volunteers participated in the experiment (3 left-handed, 8 males, average age 25.0). All recruitment, screening, and experimental procedures complied with the Declaration of Helsinki and were approved by Aston University ethics committee.

### Experimental Procedures

**Fig. 1:**
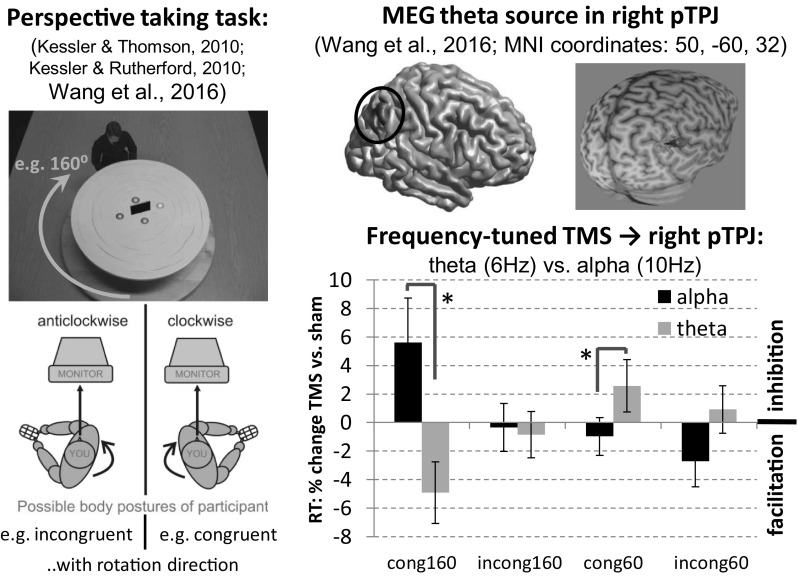
Experimental paradigm adopted from Wang et al. (2016; also Kessler and Rutherford 2010; Kessler and Thomson 2010), showing an avatar at a round table sitting at either 60° or 160° angular disparity (clock- or anticlock-wise). Participants were instructed to press the left mouse button for a “left” target (*red sphere*) and the right mouse button for a “right” target in concordance with the avatar’s perspective. Below the stimulus the “posture manipulation” is shown, where the participant’s body was either turned clock- or anticlock-wise, while the head remained straight, gazing ahead at the screen. Hence, the body-turn could either be congruent or incongruent with the direction of mental (self-) rotation into the other’s perspective (Kessler and Thomson 2010). The brain image at the *top right* shows the pTPJ source obtained for theta oscillations in Wang et al. (2016). Using Brainsight^®^ TMS neuronavigation and individual MRIs (*top far-right*), the current study employed the shown MNI coordinates as a target for 15 TMS pulses at either theta (6 Hz) or alpha (10 Hz) frequency, administered before the onset of the avatar stimulus. The *graph* at the *bottom right* shows response times (RT) as a percent-change for each TMS condition in relation to its sham baseline. *Negative values* in the *graph* indicate facilitation due to TMS entrainment compared to sham, while *positive values* reflect inhibition. *Asterisks* indicate significant t-statistics at the 5% level (for *congruent-160°* t (13) = 2.4, p = .03; for *congruent-60°* t (13) = 2.28, p < .04). *Error bars* denote the standard error of mean. (Color figure online)

Participants were required to press the left mouse button for a “left” target and the right mouse button for a “right” target in concordance with an avatar’s perspective, positioned at various angular disparities (Fig. 1). Figure 1 further shows the unique posture manipulation, which led Kessler and Thomson (2010; replicated in Kessler et al. 2014; Kessler and Rutherford 2010; Surtees et al. 2013) to conclude that visuospatial perspective taking is an embodied process: When the participant’s posture was turned towards the target viewpoint (congruent), perspective transformations were faster than when it was turned in the other direction (incongruent). Wang et al. (2016) replicated this behavioural effect in their MEG experiment and in the TMS sham condition, while dpTMS to right pTPJ abolished the posture effect.

Stimuli, conditions and experimental procedures were identical to the dpTMS experiment in Wang et al. (2016), with the only difference that instead of 2 TMS pulses administered to right pTPJ (MNI-coordinates: 50, −60, 32) during presentation of the perspective taking stimulus (Fig. 1), 15 pulses (at 90% individual motor threshold) at either theta frequency (6 Hz) or alpha frequency (10 Hz) were administered, with the final pulse ending just before stimulus onset (e.g. Hanslmayr et al. 2014). Click trains were played on all trials (sham and stimulation) at either 6 or 10 Hz, thus, participants were unable to distinguish acoustically between TMS and sham trials. However, somatosensory sensations may have still allowed for a discrimination.

Our design included four repeated measures factors: *angular disparity* (60°/160°), *posture congruence* (congruent/incongruent), *TMS condition* (stimulation/sham), and *stimulation frequency* (6 vs. 10 Hz). 320 trials were administered in total (20/condition) and conditions were presented randomly apart from posture and frequency that were blocked into 16 and 32 trial mini-blocks, respectively (see Supplementary Material for further details).

## Results and Discussion

We calculated the response time (RT) percent-change for each TMS condition in relation to its sham baseline ((sham−tms)/sham × 100) and then conducted a 3-way repeated measures ANOVA (see Supplementary Material for uncorrected data and analysis). We observed a significant interaction between frequency and angular disparity [F(1, 13) = 7, p = .02, η²_p_ = 0.351] indicating that the two entrainment frequencies had opposite effects (Fig. 1). Conforming to our hypothesis, theta facilitated visuospatial perspective taking at 160°, while alpha slowed it down, and the reverse was true at 60°. This differential effect was more pronounced for a congruent posture as revealed by post-hoc t-tests (Fig. 1) and a statistical trend for a 3-way interaction frequency × angle × posture [F(1, 13) = 3.8 p = .074, η²_p_ = 0.225]. The facilitatory effect of alpha at 60° compared to 160° is also noteworthy, suggesting that inhibition of embodied processing in pTPJ (alpha entrainment) at 60° might facilitate a visual processing strategy: At 60° the target is still visibly left or right from an egocentric perspective, thus, a perspective transformation is not strictly necessary. For discussion see Kessler and Thomson, (2010) and Wang et al. (2016).

In conclusion our results further corroborate our previous MEG finding that theta oscillations in the right pTPJ are of significance to embodied perspective transformations. As discussed in detail in Wang et al. (2016) pTPJ indeed seems to control representations of self vs. “other” (e.g., Santiesteban et al. 2012), however, we propose that the “other” might actually be the simulation of an alternative embodied self that serves as the basis for inferring another’s mental representations (e.g. their perspective).

## Electronic supplementary material

Below is the link to the electronic supplementary material.

Supplementary material 1 (DOCX 48 KB)
