# Supplementary material for: THETA-Rhythm Makes the World Go Round: Dissociative Effects of TMS Theta Versus Alpha Entrainment of Right pTPJ on Embodied Perspective Transformations
Source: Brain Topogr. 2017 Mar 3;30(5):561–4. doi: 10.1007/s10548-017-0557-z (PMC5563337; doi:10.1007/s10548-017-0557-z)
Supplement: Supplementary file 1 — Supplementary material 1 (DOCX 48 KB) [file 10548_2017_557_MOESM1_ESM.docx]

**Supplementary Material**

Experimental Procedures

Conforming to Wang et al. (2016, Expt. 2; see also Fig. 1 in main text) our design included the three repeated measures factors *angular disparity* (60°/160°), *posture congruence* (congruent/incongruent), *TMS condition* (stimulation/sham), but also included *stimulation frequency* (6 vs. 10Hz) as a fourth repeated measures factor. 20 trials were administered per condition (320 trials in total) and conditions were presented randomly apart from posture and frequency. A particular posture (e.g. turned clockwise) was adopted throughout a mini-block of 16 trials and then changed to the other posture for the next mini-block. A single TMS entrainment frequency, theta or alpha, was applied throughout a block of 32 trials (2 mini-blocks). Starting postures and frequencies were counter-balanced across participants.

In the current study we targeted the identical pTPJ site as in Wang et al., (2016; MNI-coordinates: 50, -60, 32) using Brainsight® neuronavigation and individual MRIs. Pulse intensity was 90% of the individual motor threshold, as determined by standard protocols (Rossini et al. 1994) and 15 pulses were administered at either theta frequency (6Hz) or alpha frequency (10Hz) before stimulus onset, with the final pulse ending just before stimulus onset (e.g. Hanslmayr et al. 2014). In addition we followed the rationale of Wang et al. (2016) and employed sham trials (no TMS pulses), where only acoustic clicks were administered via earphones. TMS stimulation and sham frequencies at either 6 or 10 Hz were administered in a blocked design. Click trains were played on all trials, sham and stimulation, and at the TMS stimulation frequency for a given block, i.e. at either 6 or 10 Hz. Clicks were loud enough (administered via in-ear headphones) to mask the acoustic discharge of the coil, therefore, participants were unable to distinguish acoustically between TMS and sham trials, yet, somatosensory sensations may have still allowed for a discrimination. Nevertheless, the obtained pattern of results described in the main text and in the next section rules cannot be explained by a simple frequency-discrimination or frequency–specific entrainment effect (disregarding TMS stimulation or acoustic stimulation only) as discussed below.

Results

*Uncorrected Response Times (RTs)*

Table S1 and Figure S1 report group data for each condition of the design: frequency (6 vs. 10 Hz), TMS (stimulation vs. acoustic sham), angular disparity (160 vs. 60 deg), posture congruence (congruent vs. incongruent). The data were subjected to a 4way ANOVA that revealed a significant main effect of angle (F(1, 13), p<.0001, np2 = .651), a significant 2way interaction between frequency x angle (F(1, 13), p=.017, np2 = .365), and a significant 3way interaction between frequency x angle x TMS (F(1, 13), p=.036, np2 = .296). Statistical trends (p <.1) were observed for main effects of frequency and congruence, and for the 4way interaction frequency x angle x congruence x TMS.

Table S1. Means (avg) and standard deviations (stdev) for RTs in each condition.

|  | **Alpha** |  |  |  |  |  |  |  |
| --- | --- | --- | --- | --- | --- | --- | --- | --- |
|  | **160 cong TMS** | **160 cong SHAM** | **160 inc TMS** | **160 inc SHAM** | **60 cong TMS** | **60 cong SHAM** | **60 inc TMS** | **60 inc SHAM** |
| ***avg*** | 772.18 | 734.39 | 752.39 | 757.71 | 576.68 | 584.57 | 573.36 | 598.07 |
| ***stdev*** | 223.14 | 181.06 | 172.52 | 164.31 | 94.01 | 87.82 | 76.73 | 103.36 |
|  | **Theta** |  |  |  |  |  |  |  |
|  | **160 cong TMS** | **160 cong SHAM** | **160 inc TMS** | **160 inc SHAM** | **60 cong TMS** | **60 cong SHAM** | **60 inc TMS** | **60 inc SHAM** |
| ***avg*** | 709.61 | 760.04 | 758.14 | 771.89 | 610.07 | 596.21 | 619.32 | 615.75 |
| ***stdev*** | 154.55 | 186.31 | 175.48 | 184.90 | 110.53 | 95.40 | 107.37 | 100.60 |

The significant 2way interaction between frequency and angle (and the trend for a main effect of frequency) could indicate that a certain level of frequency-specific effects might occur due to acoustic entrainment (i.e., disregarding TMS stimulation or sham). However, active TMS stimulation had a significant modulating effect on top of the 2way interaction in form of a significant 3way interaction (and a trend for a 4way interaction including posture congruence). Accordingly, for a sham-only analysis the 2way interaction between frequency x angle (as well as the 3way interaction with posture congruence) did not reach significance (p=.7 and p=.6, respectively), while for a stimulation-only analysis it did (F(1,13)=8.3, p=.013, η²_p_=.391; and a trend for the 3way interaction also observed: F(1,13)=4.5, p=.053, η²_p_=.259), suggesting that the original 2way interaction (frequency x angle) was mainly driven by the active stimulation condition, conforming to the significant 3way interaction. In conclusion, acoustic entrainment can be ruled out as the main explanation of our results, highlighting frequency-tuned TMS stimulation as the more relevant factor. This further justifies the use of a percentage-change analysis (stimulation vs. sham baseline) reported in the main text.


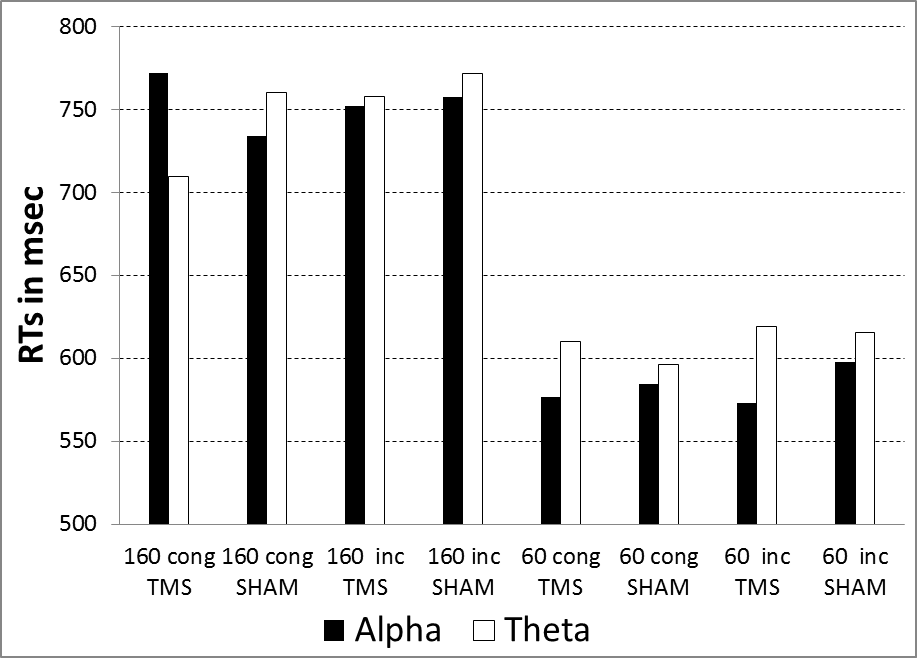


Figure S1: Means per condition for the full design. For standard deviations see Table S1.

*Accuracy data (percent correct responses)*

Table S2. Means (avg) and standard deviations (stdev) for percent of correct responses per condition.

|  | **Alpha** |  |  |  |  |  |  |  |
| --- | --- | --- | --- | --- | --- | --- | --- | --- |
|  | **160 cong TMS** | **160 cong SHAM** | **160 inc TMS** | **160 inc SHAM** | **60 cong TMS** | **60 cong SHAM** | **60 inc TMS** | **60 inc SHAM** |
| ***avg*** | 94.83 | 92.78 | 91.44 | 93.99 | 96.61 | 99.20 | 96.96 | 96.64 |
| ***stdev*** | 7.48 | 6.93 | 8.91 | 6.31 | 5.40 | 2.06 | 8.37 | 6.32 |
|  | **Theta** |  |  |  |  |  |  |  |
|  | **160 cong TMS** | **160 cong SHAM** | **160 inc TMS** | **160 inc SHAM** | **60 cong TMS** | **60 cong SHAM** | **60 inc TMS** | **60 inc SHAM** |
| ***avg*** | 96.16 | 94.70 | 94.82 | 94.61 | 99.22 | 98.57 | 97.14 | 97.86 |
| ***stdev*** | 4.91 | 6.04 | 7.24 | 6.93 | 1.98 | 3.06 | 5.08 | 4.69 |

References

Hanslmayr S, Matuschek J, Fellner M-C (2014) Entrainment of prefrontal beta oscillations induces an endogenous echo and impairs memory formation *Current Biology* 24:904-909

Rossini PM et al. (1994) Non-invasive electrical and magnetic stimulation of the brain, spinal cord and roots: basic principles and procedures for routine clinical application. *Report of an IFCN committee Electroencephalography and clinical neurophysiology* 91:79-92

Wang H, Callaghan E, Gooding-Williams G, McAllister C, Kessler K (2016) Rhythm makes the world go round: An MEG-TMS study on the role of right TPJ theta oscillations in embodied perspective taking *Cortex* 75:68-81
